# Supplementary material for: Balanced Force Field ff03CMAP Improving the Dynamics Conformation Sampling of Phosphorylation Site
Source: Int J Mol Sci. 2022 Sep 25;23(19):11285. doi: 10.3390/ijms231911285 (PMC9569523; doi:10.3390/ijms231911285)
Supplement: Supplementary file 1 [file ijms-23-11285-s001.zip › ijms-1837145-supplementary.pdf]

International Journal of
*Molecular Sciences*

MDPI

*Supplementary*

# Balanced Force Field ff03CMAP Improving the Dynamics Conformation Sampling of Phosphorylation Site

**Bozitao Zhong [1,†], Ge Song [1,†] and Hai-Feng Chen [1,2,*]**

[1] State Key Laboratory of Microbial Metabolism, Joint International Research Laboratory of Metabolic and Developmental Sciences, Department of Bioinformatics and Biostatistics, National Experimental Teaching Center for Life Sciences and Biotechnology, School of Life Sciences and Biotechnology, Shanghai Jiao Tong University, Shanghai 200240, China
[2] Shanghai Center for Bioinformation Technology, Shanghai 200240, China
* Correspondence: haifengchen@sjtu.edu.cn; Tel./Fax: +86-21-34204073
† These authors contributed equally to this work.

**Citation:** Zhong, B.; Song, G.; Chen, H.-F. Balanced Force Field ff03CMAP Improving the Dynamics Conformation Sampling of Phosphorylation Site. *Int. J. Mol. Sci.* **2022**, *23*, 11285. https://doi.org/10.3390/ijms231911285

Academic Editors: Christo Z. Christov and Tatyana Karabencheva-Christova

Received: 12 July 2022
Accepted: 11 September 2022
Published: 25 September 2022

**Publisher's Note:** MDPI stays neutral with regard to jurisdictional claims in published maps and institutional affiliations.

**Copyright:** © 2022 by the authors. Licensee MDPI, Basel, Switzerland. This article is an open access article distributed under the terms and conditions of the Creative Commons Attribution (CC BY) license (https://creativecommons.org/licenses/by/4.0/).

**Table S1.** Experimental measurements used in this work.

| System | Experimental measurements |
|---|---|
| **Peptide** | |
| GpSG | |
| GpTG | Backbone Cα, Cβ, C, N, Hα and HN chemical shifts |
| GpYG | |
| **Disordered Protein** | |
| TF | Backbone Cα, Cβ, N, Hα and HN chemical shifts |
| Vg | Backbone N, Hα and HN chemical shifts |
| **Folded Protein** | |
| β3 | Backbone Cα, Hα and HN chemical shifts |
| αM | Backbone Cα, Cβ, C, N, Hα and HN chemical shifts |
| Ets1 | Backbone Cα, Cβ, C, N, Hα and HN chemical shifts |
| p-Ubiquitin | Backbone Cα, Cβ, C, N, Hα and HN chemical shifts |
| **Complex** | |
| SH2 | Backbone Cα, Cβ, C, N, Hα and HN chemical shifts |
| TrkB | Backbone Cα, Cβ, and Hα chemical shifts |

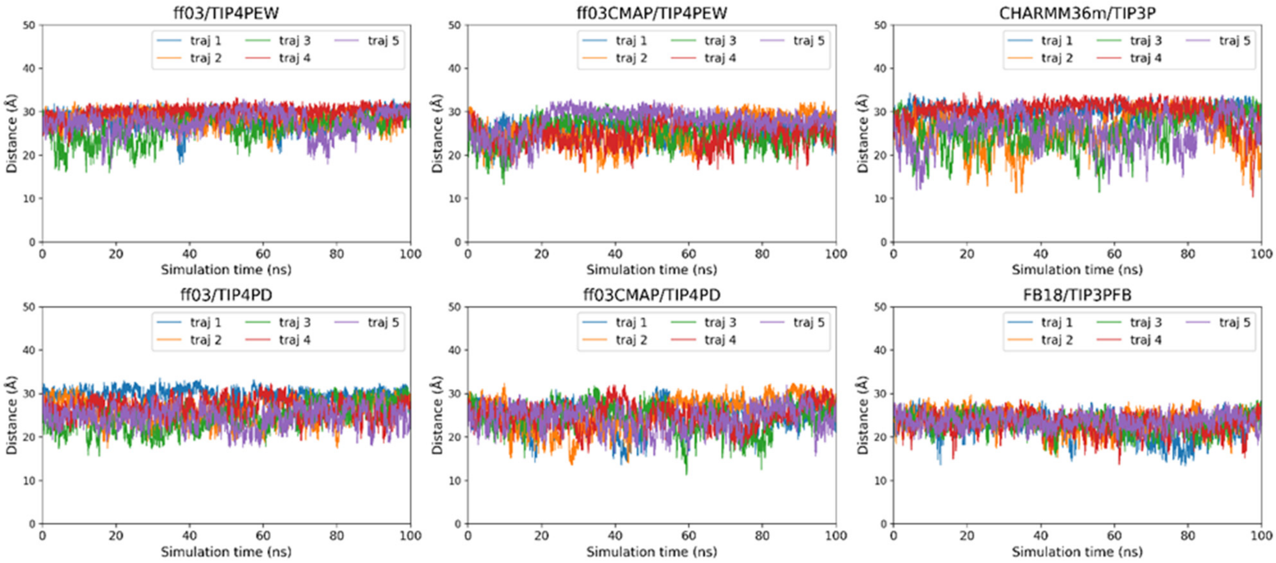


**Figure S1.** Minimum distance between periodic images v.s. simulation time of the disordered TF protein. Five parallel trajectories of each force field are presented in each plot. All plots support that the distance is always greater than the cutoff value (8 Å). .

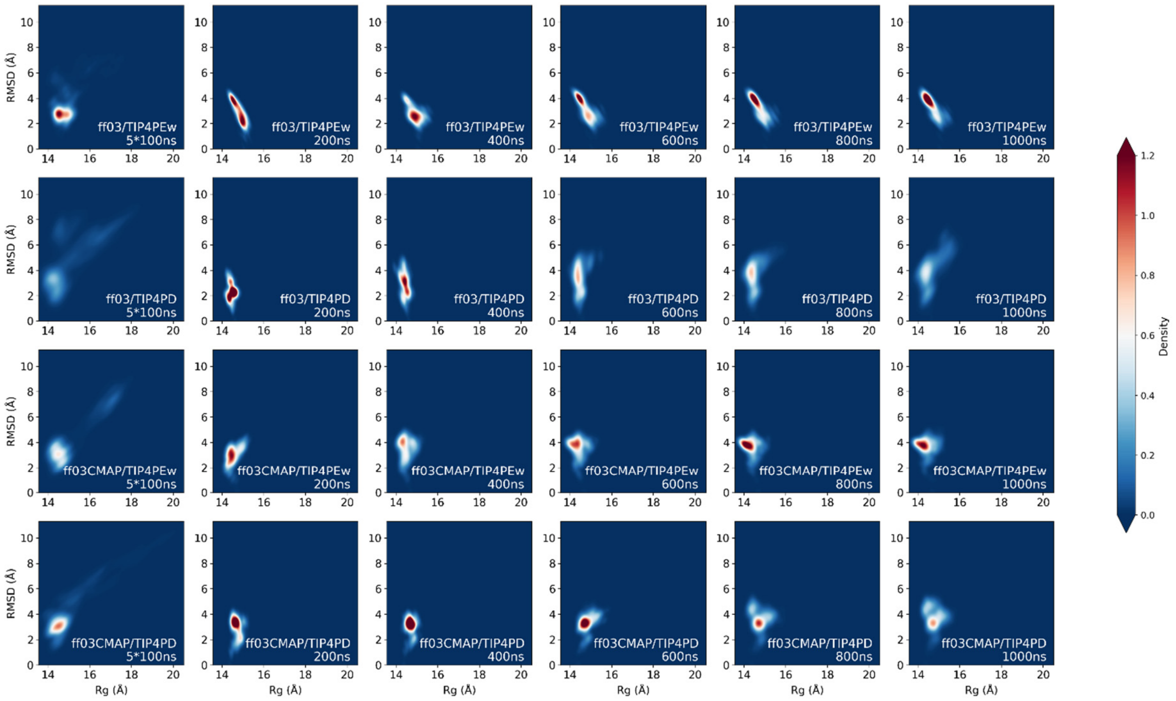


**Figure S2.** Comparison of free energy landscape of Ets-1 system with 5 parallel 100 ns simulation and 1 long 1000 ns simulation. Left column: simulation result of 5*100 ns simulation of 4 simulated systems. Other columns: simulation of 1000 ns simulation of 4 simulated systems within first 200/400/600/800/1000 ns. Results showed that 5 parallel 100 ns simulation has larger sampling size than 1 long 1000 ns simulation.

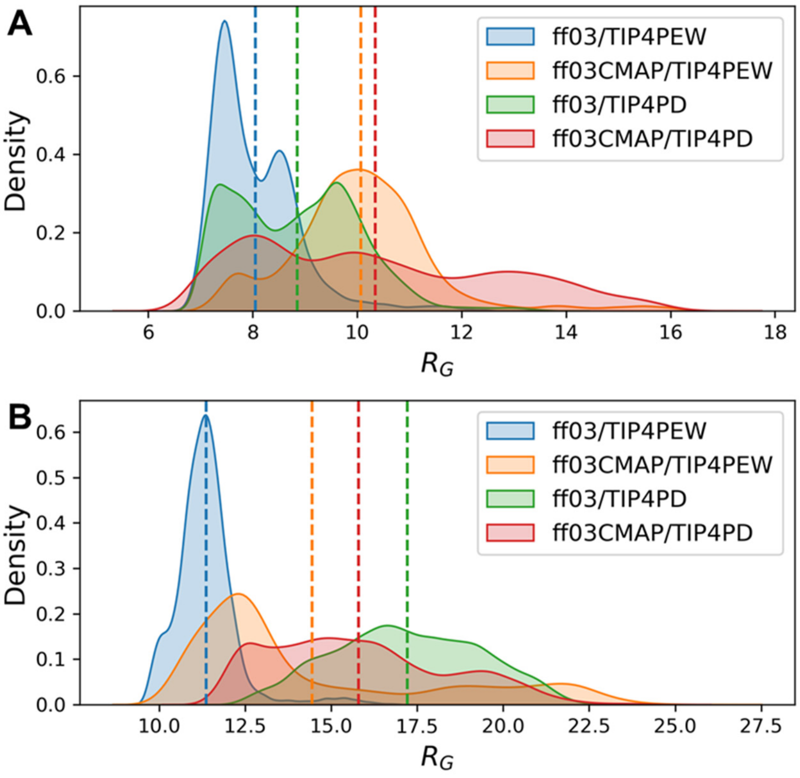


**Figure S3.** Radius of gyration ($R_G$) distribution of simulated phosphorylated disordered proteins. Distribution of radius of gyration of tissue factor cytoplasmic tail (A) and vitellogenin (B) is presented. Results showed that ff03CMAP has relative higher mean $R_G$ then ff03, and

ff03CMAP/TIP4PD usually have wild distribution. Colored dashed line represents the mean of $R_G$, and solid line and shade present the probability density curve estimated by Gaussian kernel.

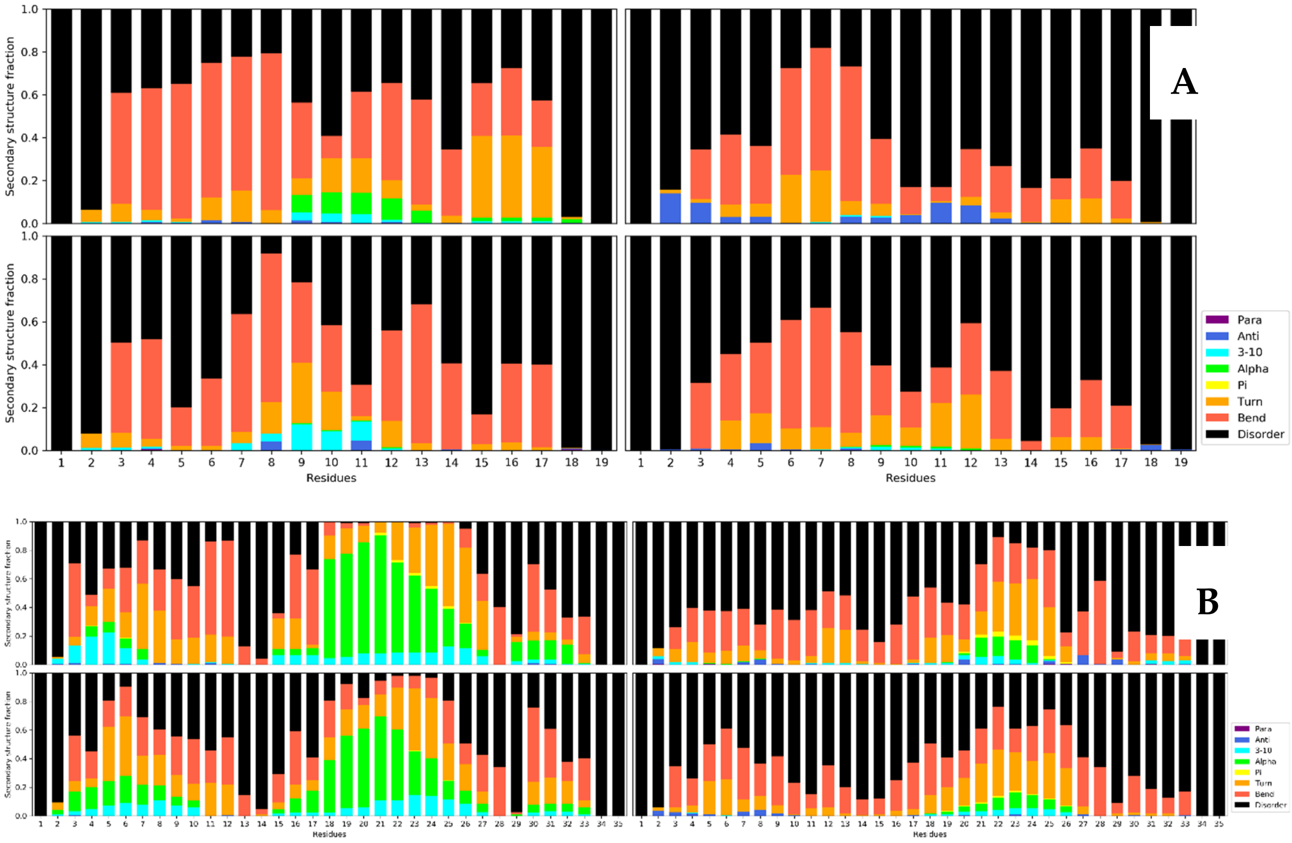


**Figure S4.** Secondary structure of simulated phosphorylated disordered proteins. A:2CEF. B: 2LID.

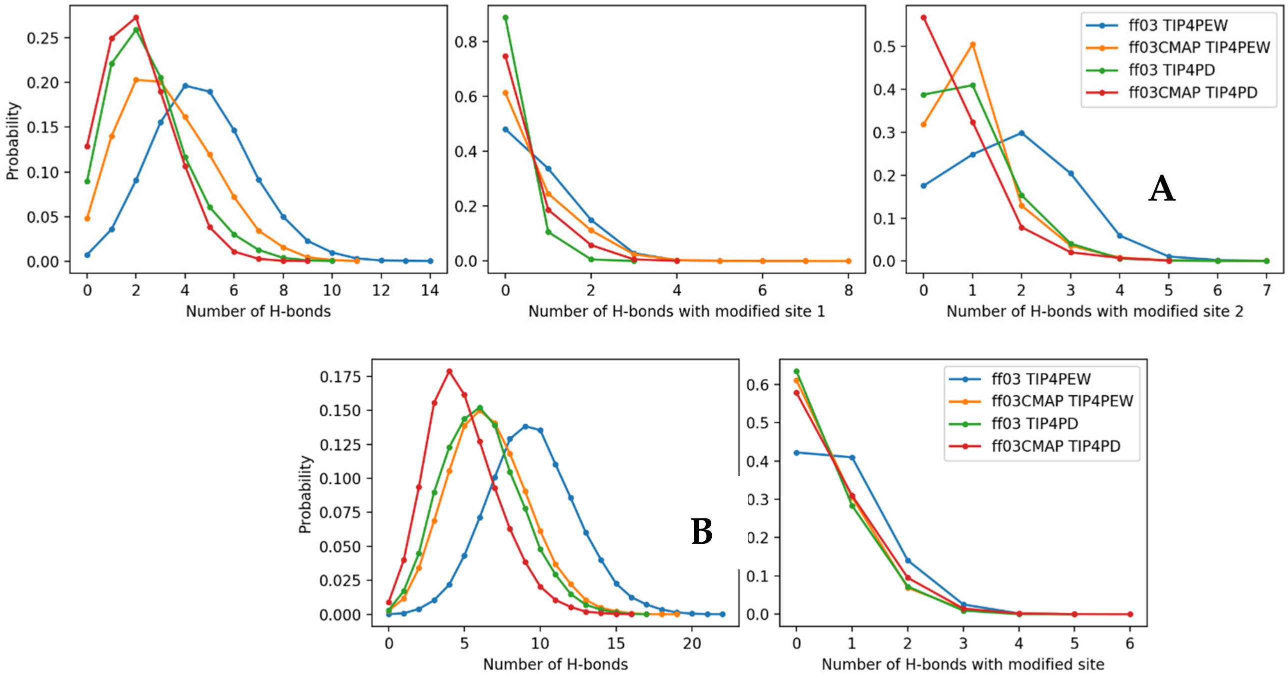


**Figure S5.** Hydrogen bond of simulated phosphorylated disordered proteins. A:2CEF. B: 2LID.

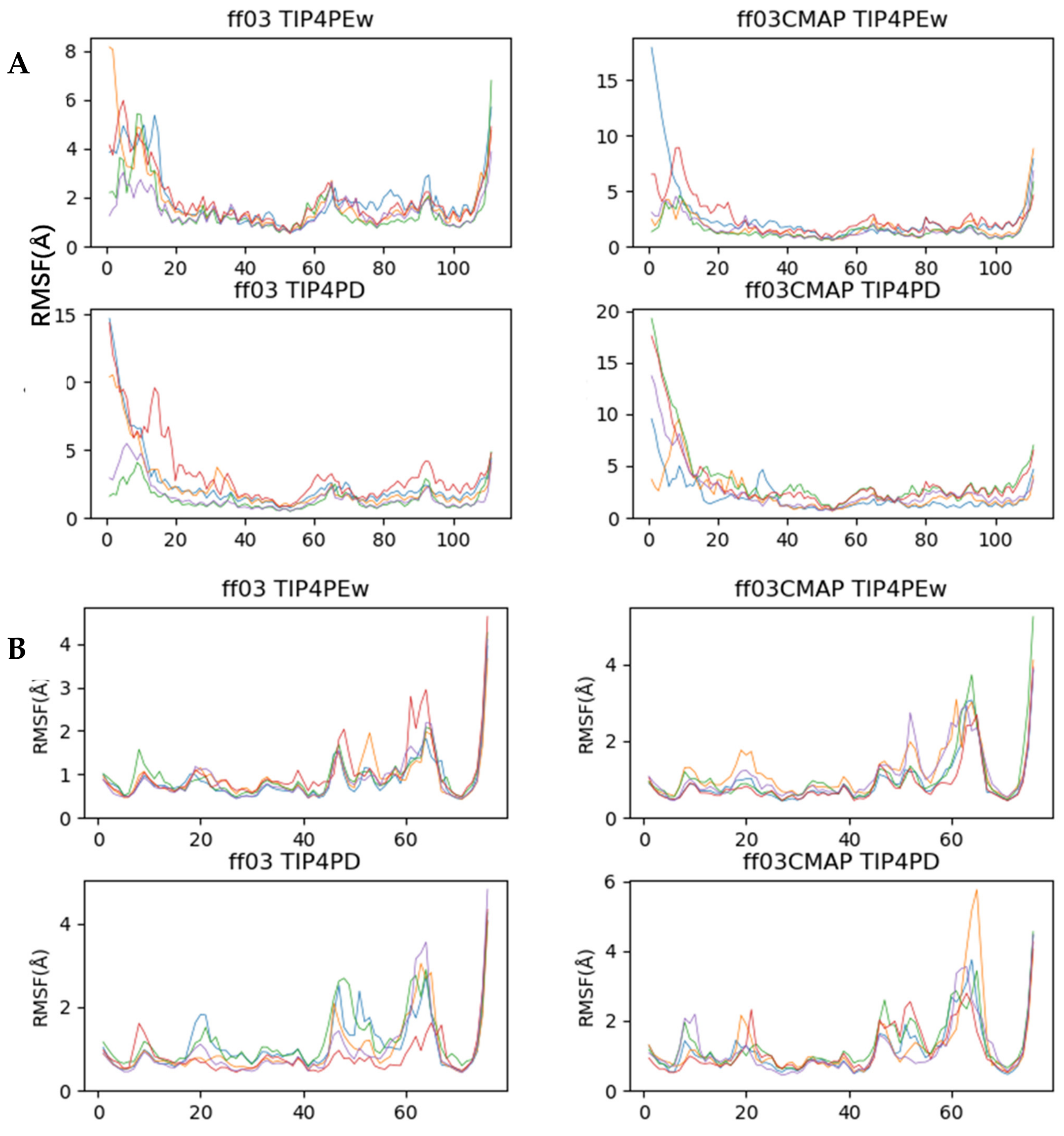


**Figure S6.** RMSF of simulated Ets-1 and p-Ubiquitin. A: Ets-1. B: p-Ubiquitin.

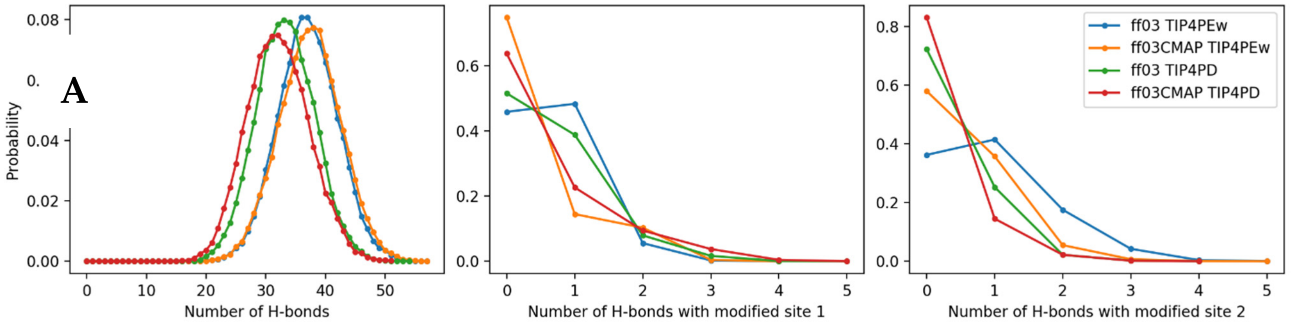

**B**

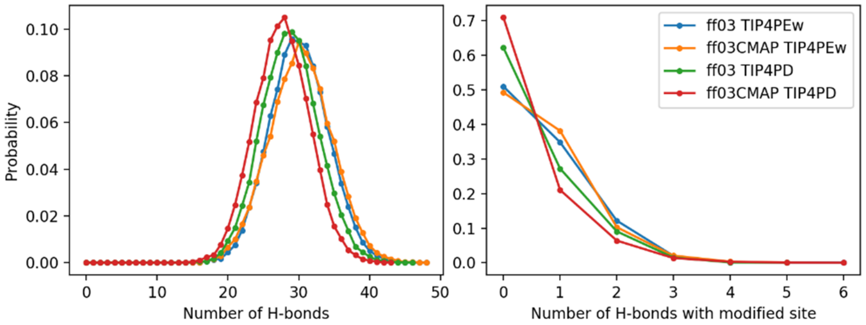


**Figure S7.** Hydrogen bond in phosphorylation site of simulated Ets-1 and p-Ubiquitin. A: Ets-1 2KMD. B: p-Ubiquitin 5XK4.

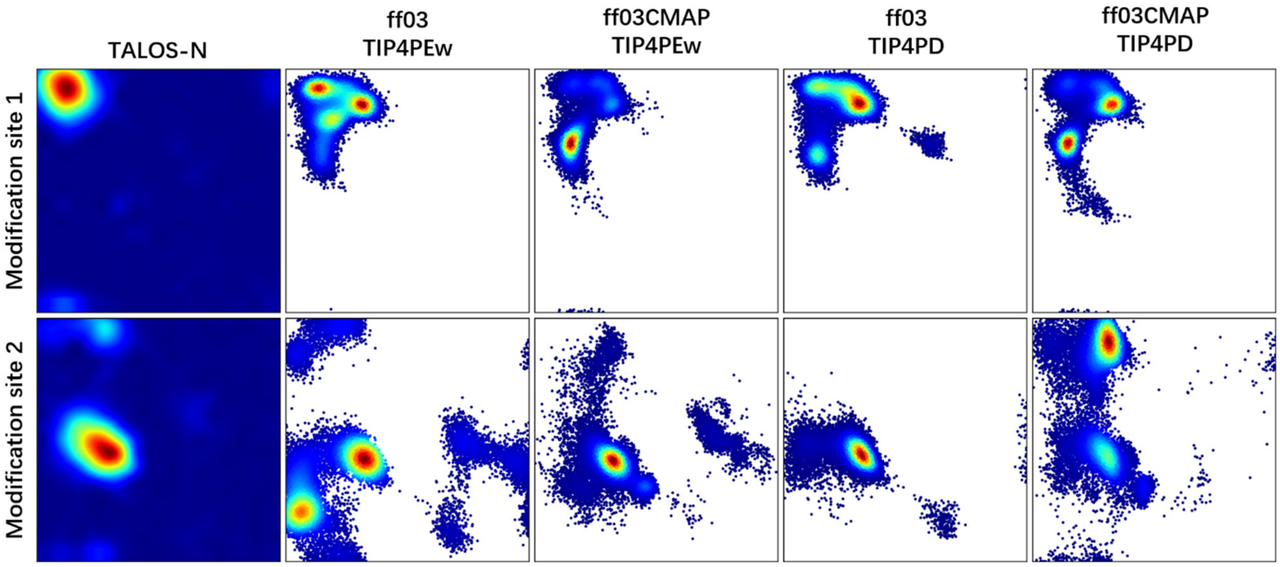


**Figure S8.** Ramachandran plot of phosphorylated sites in Ets-1 simulation.

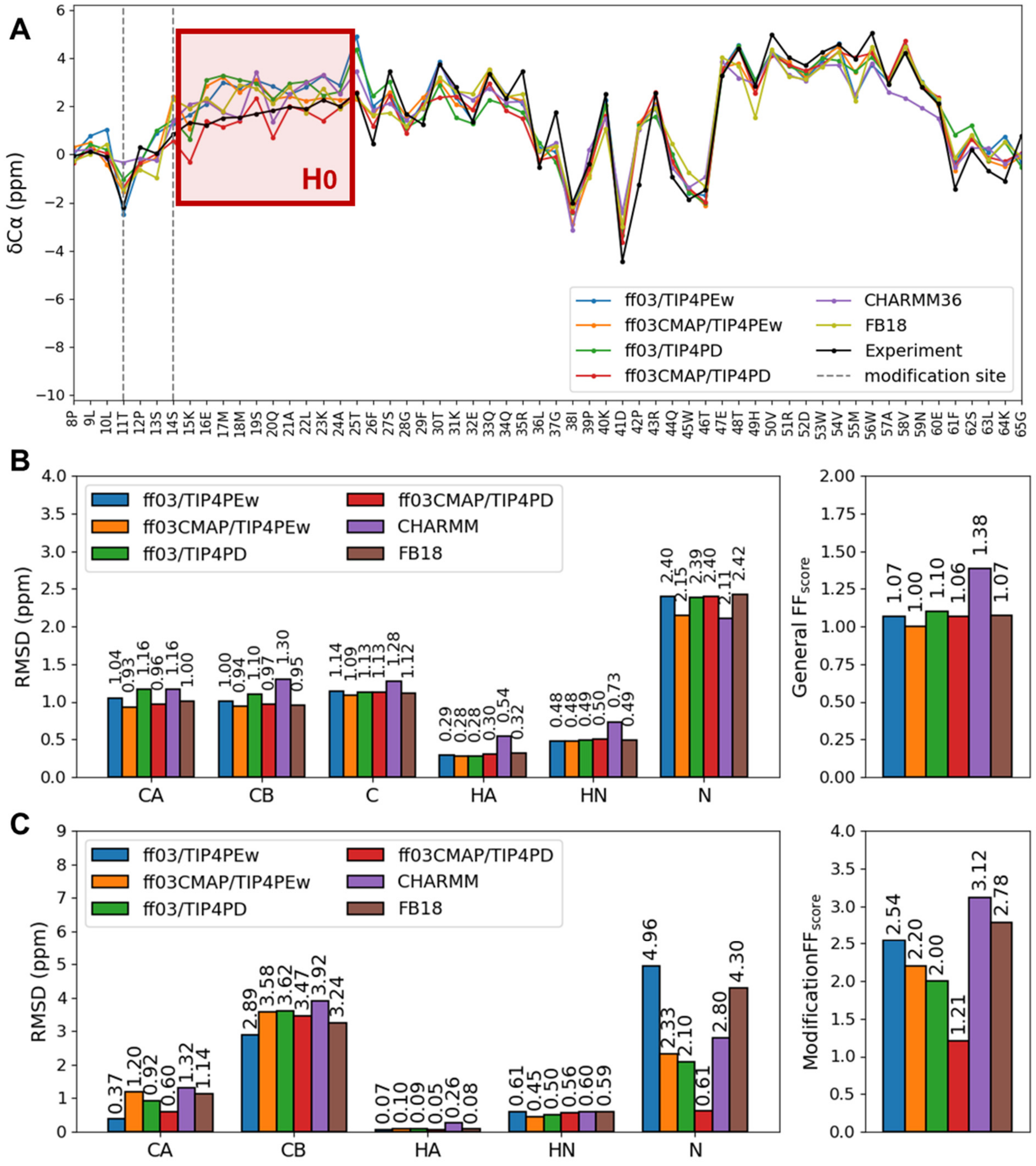


**Figure S9.** Comparison of ff03, ff03CMAP with CHARMM36m and FB18 force fields in Ets-1 (PDB: 2KMD) system. Simulation for each system is 5*100 ns. (A) present the secondary chemical shift of CA atom in Ets-1, H0 region is highlighted. (B) showed the RMSD between simulated and experimental chemical shift of all residues, which (C) showed the RMSD between simulated and experimental chemical shift of phosphorylated residues. Besides, (B) and (C) also showed the general FFscore and ModificationFFscore. In this case, *ff03CMAP/TIP4PD* still performed best among all 6 systems in phosphorylated residues when comparing more force fields.

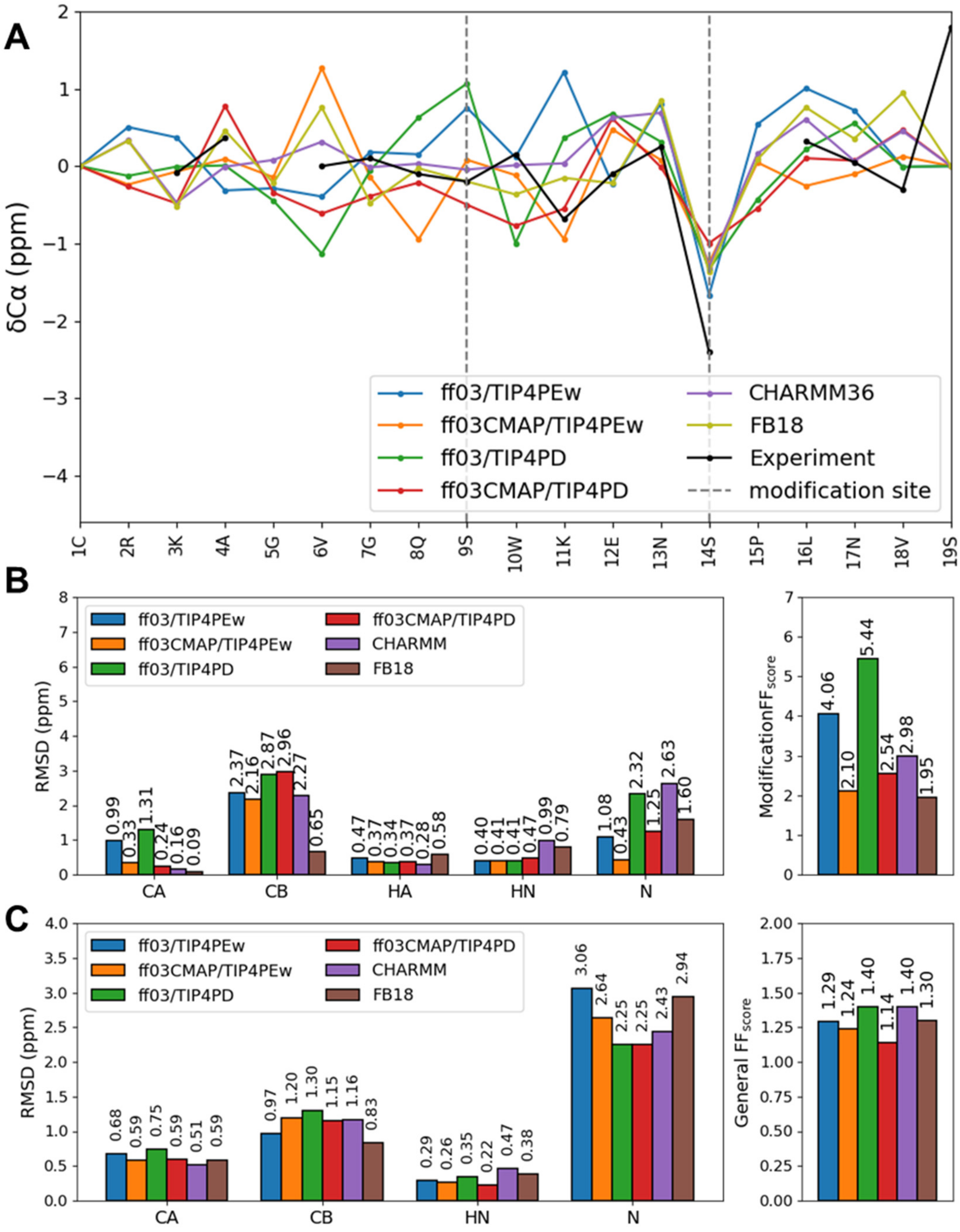


**Figure S10.** Comparison of ff03, ff03CMAP with CHARMM36m and FB18 force fields in Tissue Factor (TF, PDB: 2CEF) system. Simulation for each system is 5*100 ns. (A) present the secondary chemical shift of CA atom in TF. (B) showed the RMSD between simulated and experimental chemical shift of all residues, which (C) showed the RMSD between simulated and experimental chemical shift of phosphorylated residues. Besides, (B) and (C) also showed the general FFscore and ModificationFFscore. In this case, *ff03CMAP/TIP4PD* still has relative low ModificationFFscore and lowest General FFscore.
